# Mapping quantitative trait loci responsible for resistance to Bakanae disease in rice

**DOI:** 10.1186/s12284-016-0117-2

**Published:** 2016-09-13

**Authors:** R. Abdul Fiyaz, Ashutosh K. Yadav, S. Gopala Krishnan, Ranjith K. Ellur, Bishnu M. Bashyal, Nitasha Grover, Prolay K. Bhowmick, M. Nagarajan, K. K. Vinod, Nagendra K. Singh, Kumble V. Prabhu, Ashok K. Singh

**Affiliations:** 1 Division of Genetics, ICAR-Indian Agricultural Research Institute, New Delhi, 110012 India; 2 Division of Plant Pathology, ICAR-Indian Agricultural Research Institute, New Delhi, 110012 India; 3 Rice Breeding and Genetics Research Centre, ICAR-Indian Agricultural Research Institute, Aduthurai, 612101 India; 4 ICAR-National Research Centre on Plant Biotechnology, New Delhi, 110012 India; 5 Present address: ICAR-Indian Institute of Rice Research, Hyderabad, 500030 India

**Keywords:** Rice, Bakanae, Foot rot, *Fusarium fujikuroi*, QTL mapping, Resistance

## Abstract

**Background:**

Bakanae or foot rot disease caused by *Fusarium fujikuroi* [teleomorph: *Gibberella fujikuroi* (Sawada) Ito] is emerging as a serious disease in rice. The disease causes both quantitative and qualitative losses to the grains under the field conditions. Breeding for resistance to Bakanae disease is a promising strategy to manage this emerging disease. In this study, we used a population of 168 F_14_ recombinant inbred lines (RILs) derived from two *indica* rice parents Pusa 1342, a highly resistant variety and Pusa Basmati 1121, a highly susceptible variety to map quantitative trait loci (QTLs) governing resistance against Bakanae disease.

**Results:**

The disease reaction of 168 F_14_ RILs were measured on the seedlings inoculated using *Fusarium fujikuroi* culture using high-throughput screening protocol under glasshouse conditions. Utilizing inclusive composite interval mapping, three QTLs governing resistance to Bakanae were identified, namely *qBK1.1*, *qBK1.2* and *qBK1.3* which accounted 4.76, 24.74 and 6.49 % of phenotypic variation, respectively. The major effect QTL designated *qBK1.2* was mapped in 0.26 Mb region between RM5336 and RM10153. A total of 55 annotated genes were identified within the identified QTL region *qBK1.2*.

**Conclusions:**

The novel QTLs identified in this study are useful resource for efficiently breeding rice cultivars resistant to Bakanae disease. This is the first report on identification of QTLs governing resistance against Bakanae in rice using inclusive composite interval mapping strategy in a RIL population.

## Background

Rice is one of the most important staple food crops in the world which is grown under diverse ecological conditions and thus gets exposed to different biotic and abiotic stresses. Among the biotic stresses, insect pests and diseases caused by bacteria, fungi, nematodes and viruses are the major factors affecting the rice production. Among the potentially important diseases of contemporary importance, Bakanae or foot rot disease, caused by *Fusarium fujikuroi* (Nirenberg), [teleomorph: *Gibberella fujikuroi* (Sawada) Ito] has emerged as a disease of major concern (Bashyal et al. 2016). The disease can cause upto 70 % yield loss and impairs the grain quality as well, under the field conditions (Fiyaz et al. 2014). Bakanae disease of rice occurs widely throughout Asia and sporadically in other areas of rice production (Sun and Snyder 1981; Webster and Gunnell 1992). The term ‘Bakanae’ is of Japanese origin meaning ‘bad’, ‘naughty’ or ‘foolish’ seedling, indicating the unusual early elongation of seedlings due to the production of gibberellin on infection process. The typical symptom also includes yellowing of the affected seedlings. The fungus produces both gibberellins and fusaric acid, and the seedling elongation is attributed to the former and stunting to the later. The type of symptoms and the severity of the disease are dependent on the quantity of the two metabolites produced, which varies with different strains of the fungus and the resistance levels of the host. Since the pathogen is both seed-borne and soil-borne, infection may occur either by sowing infected seeds in non-infested fields or by sowing uninfected seeds in infested fields or by sowing infected seeds in infested fields. Generally, the seed-borne inoculum provides initial foci for secondary infection. Under favourable environmental conditions, infected plants in different foci have the capacity to produce numerous conidia that subsequently infect proximate healthy plants, which results in yield loss (Rosales and Mew 1997).

The pathogen has a wide host range and is widespread throughout the world. On rice, *F. fujikuroi* (*F. moniliforme*) induces several symptoms such as seedling elongation, foot and seedling rot, grain discoloration and sterility (Ou 1985). In older plants, the roots, crowns, stems, leaf sheaths and panicles can be infected. The fungus was reported in 1919 as *Lisea fujikuroi* Sawada, which was renamed in 1931 to *Gibberella fujikuroi* (Ito and Kimura 1931). The asexual stage was reported as *Fusarium moniliforme* (Sun and Snyder 1981). Rice plants after transplanting may also be infected, resulting in weak tillering and poor grain filling (Ou 1985; Jeff 2001). Disease at a later stage usually causes a yield loss of ~ 10–20 %, and under severe infection, the loss could go higher than 70 % (Ito and Kimura 1931; Ou 1985; Rood 2004). In recent times, use of new methods for raising seedlings, especially growing in seed boxes for mechanical transplanting (Rosales and Mew 1997) and dry seed-bed raising for hybrid rice, have favoured conditions for several minor diseases, that are not considered serious under open field nurseries. Among these, Bakanae disease is frequently encountered and has become more and more serious (Li and Luo 1997; Yang et al. 2003) leading to outbreaks in many countries like Japan, Korea and is becoming a serious threat in some rice growing regions of India and Philippines (Cumagun et al. 2011, Bashyal et al. 2014; Fiyaz et al. 2014).

Basmati is the specialty rice of India which fetches premium price in the international market for its unique cooking quality characteristics and aroma. ICAR-Indian Agricultural Research Institute (ICAR-IARI), New Delhi has developed the world’s longest cooked kernel Basmati rice variety, Pusa Basmati 1121 (PB 1121), which alone occupies > 65 % (1.35 mha.) of the total Basmati area in India, with an annual foreign exchange earning of ~ $ 4.0 billion (Singh et al. 2011). The variety is highly suited to the low input conditions, hence fits well in organic cultivation. It matures in 145 days and yields 45–50 q/ha. PB 1121 is an exquisite Basmati variety known for its extra-long slender grain, exceptionally high kernel length elongation on cooking (up to 22 mm) with an elongation ratio of 2.5, good volume expansion of more than four times, intermediate amylose content and strong aroma. Despite the aforesaid advantages, PB 1121 is highly susceptible to various diseases and pests among which Bakanae or foot rot disease has emerged as a major concern. Recently, there have been outbreaks of Bakanae disease in other Basmati rice varieties such as CSR-30, Pusa Basmati 1509 and Pusa Basmati 6 (Bashyal et al. 2016). Although Bakanae can be managed to a certain extent using chemical fungicides (Iqbal et al. 2011) through seed treatment and soil amendment, more sustainable solution is to impart genetic resistance to the disease. There are varying level of genotype response to this disease in rice, but so far there has been limited work to identify genes governing resistance to this disease. Therefore, the present study was carried out with the objective of identifying QTLs governing resistance to Bakanae disease of rice using a RIL population.

## Results

### Phenotypic Variation in Parents and the RIL Population

**Fig. 1:**
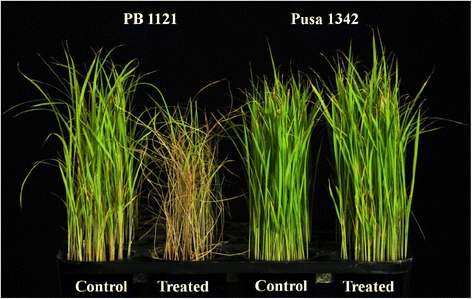
Seedling mortality of PB 1121 as against Pusa 1342 due to Bakanae disease after 15 days of inoculation

Significant phenotypic differences were detected between the two parents for Bakanae disease reaction (Fig. 1). The inoculated seeds of the genotype PB 1121, exhibited increased seedling elongation as compared to uninoculated seeds of PB 1121 (Table 1). Under inoculated conditions, the mean seedling height of PB 1121 was 24.0 ± 0.0 cm which was significantly higher than in the uninoculated control (20.7 ± 0.4 cm). However, in the resistant genotype Pusa 1342, there was no significant increase in mean seedling height under inoculated condition (12.9 ± 0.2) as compared to uninoculated condition (12.3 ± 0.5).

**Table 1:** Bakanae reaction of the two contrasting parents Pusa 1342 and PB1121 and their derived recombinant inbred population

Trait	Uninoculated		Inoculated		Inoculated RILs	
	Pusa 1342	PB 1121	Pusa 1342	PB 1121	Range	Mean
Seedling mortality (%)	0.0	0.0	2.3	99.3	0.0-100.0	32.6
Seedling height (cm) 15 DAS	12.3 ± 0.5	20.7 ± 0.4	12.9 ± 0.2	24.0 ± 0.0	-	-

*DAS* days after sowing

**Fig. 2:**
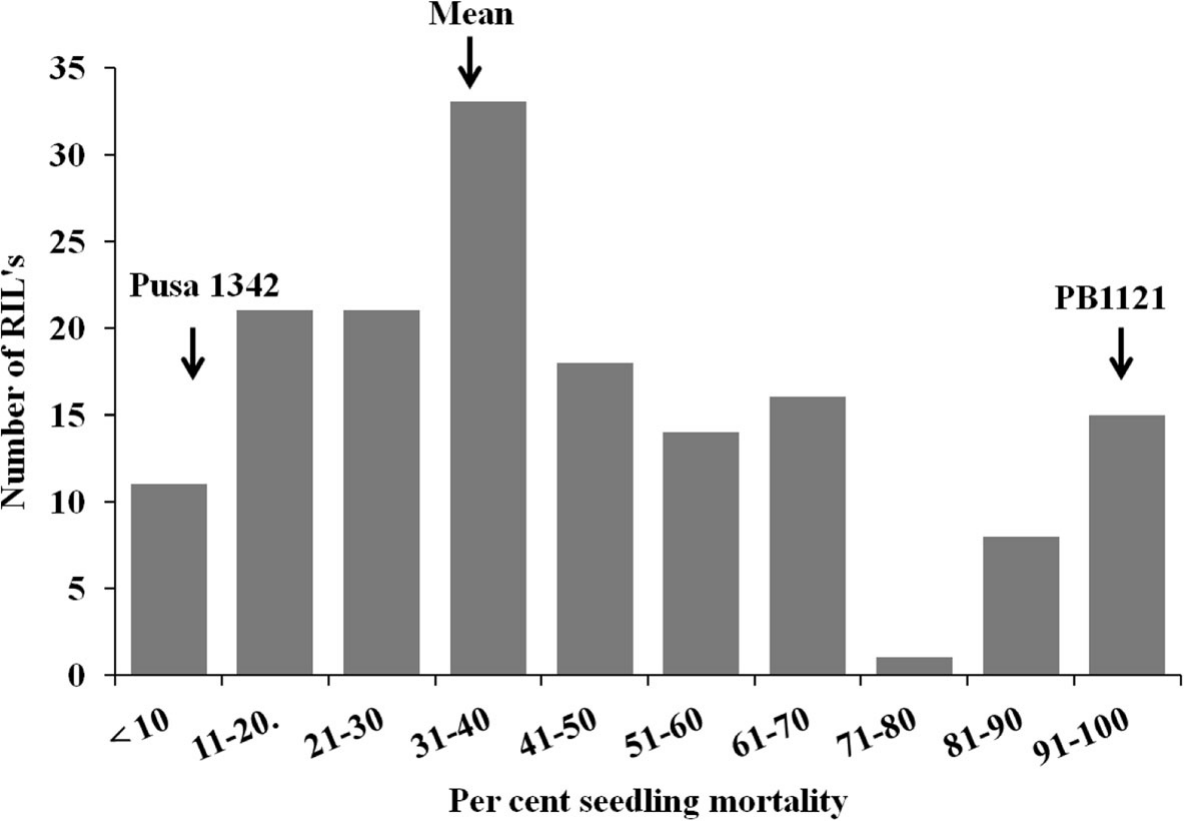
Frequency distribution of percent seedling mortality due to Bakanae infection among 168 F_14_ individuals derived from the cross (PB1121/ Pusa 1342)

Under uninoculated conditions, both the parents and the RILs had complete survivability. However, under inoculated conditions, significant phenotypic differences for seedling mortality were observed among the parents and the RILs (Table 2). The susceptible parent PB1121 and the resistant parent Pusa 1342 showed seedling mortality of 99.3 and 2.3 %, respectively. Further, among the RILs, disease reaction ranged from no mortality to 100 % mortality with very high broad sense heritability (99.97 %). The frequency distribution of disease reaction in the RILs showed higher frequency of resistant plants (less than mean mortality %) than the susceptible plants (Fig. 2).

**Table 2:** Analysis of variance (ANOVA) for percent seedling mortality under Bakanae infection

Source	df	Mean squares	Variance ratio	Probability
Replications	5	0.34	0.254	0.038
Genotypes	169	4738.70	3458.9	<0.001
Error	845	1.37		

*df* degrees of freedom, Environmental variance, σ^2^e = 1.37; phenotypic variance, σ^2^p = 4738.70; genotypic variance, σ^2^g = 4737.33; heritability (broad sense) = 99.97 %; Coefficient of variation (%) = 3.56

### Genotyping of RILs and Construction of Molecular Genetic Map

Identification of sufficient number of markers revealing polymorphism among the parental lines is a prerequisite for the construction of a genetic linkage map. In this study, a genome wide parental polymorphism survey was carried out between parents (Pusa Basmati 1121 and Pusa 1342) using a total of 732 SSR markers spanning 12 rice chromosomes (http://www.gramene.org/) and 119 SSR markers were found polymorphic (Table 3). The average per cent polymorphism between PB1121 and Pusa 1342 was low (13.98 %). The segregation distortion was analysed for all the 119 SSR loci using *χ*^2^ test, and 15 markers that deviated significantly from the expected 1:1 ratio at 5 % probability level (*χ*^2^ > 10.5) were eliminated from further analysis and the markers showing normal Mendelian segregation distributed over all the 12 rice chromosomes were used for the construction of molecular linkage map using QTL IciMapping software (Meng et al. 2015).

**Table 3:** Details of the linkage map constructed using Pusa 1342/ PB1121 RIL population

Chromosome	Total number of markers	Number of Polymorphic markers*	Polymorphism %	Map length (cM)	Marker density (cM/marker)
1	71	22	30.99	403.52	18.34
2	60	08	13.33	153.26	19.15
3	63	06	9.52	115.26	19.25
4	81	13	16.05	218.72	16.82
5	41	04	9.76	65.71	16.42
6	81	08	9.88	56.87	7.10
7	45	03	6.67	55.25	18.41
8	71	11	15.49	121.45	11.04
9	49	04	8.16	52.85	13.21
10	38	07	18.42	91.5	13.07
11	81	08	9.88	49.72	6.21
12	51	10	19.61	140.114	14.01
Total	732	104	13.98	1524.22	14.42

*cM* centimorgans, *excluding 15 markers that were showing segregation distortion

The linkage map covering a total length of 1524.22 cM was constructed using 104 SSR markers, with an average coverage of 14.42 cM per marker. Relatively, more number of polymorphic markers were found on chromosome 1 with a polymorphism of 30.99 %, while, lowest level of polymorphism was found in chromosome 7 (6.67 %). Chromosome wise marker density varied from 6.21 cM/marker in chromosome 11 to 19.25 cM/marker on chromosome 3 (Table 3).

### QTL Mapping for Bakanae Resistance

**Fig. 3:**
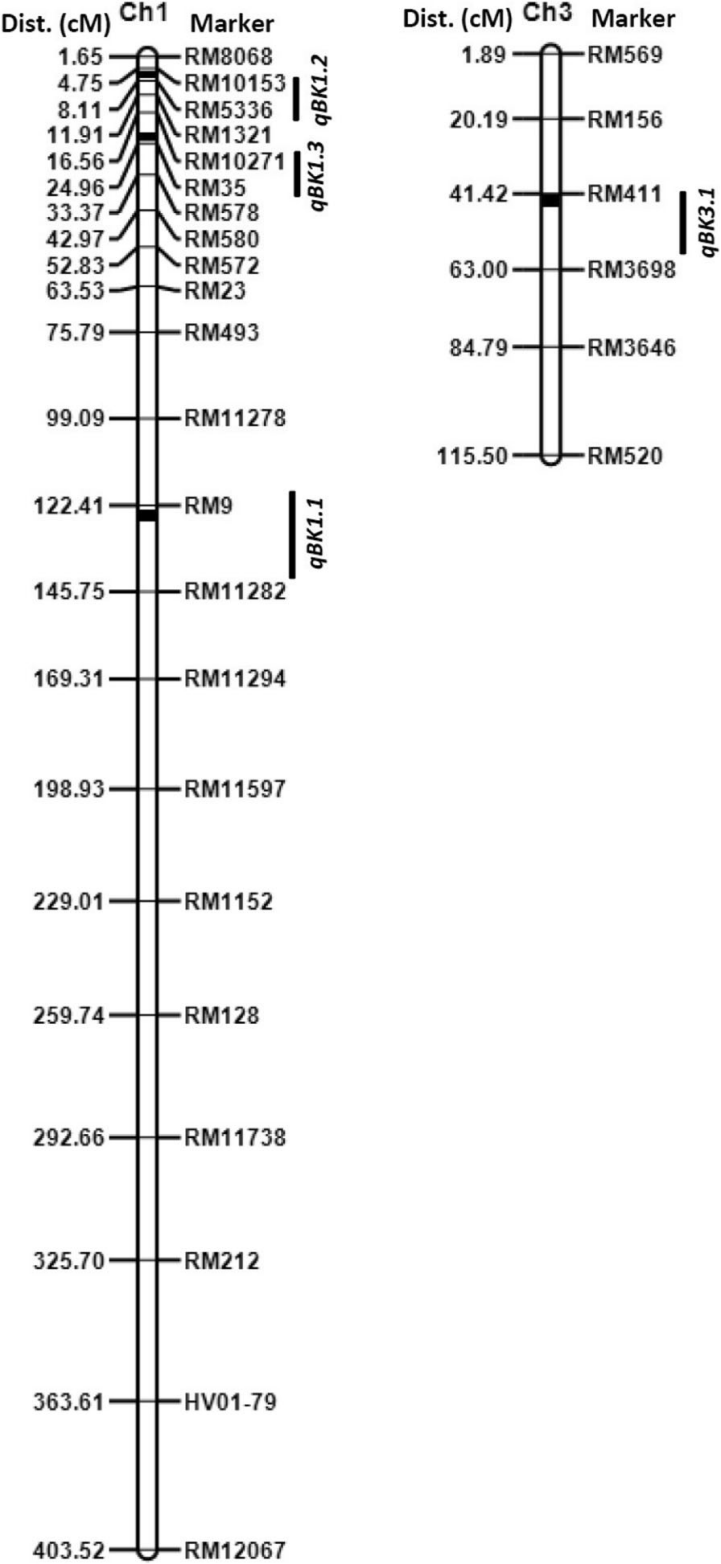
Molecular genetic map of rice along with positions of quantitative trait loci (QTLs) for Bakanae disease resistance in rice. Only those portions of the linkage maps where QTLs were detected are shown

The major objective of the present study was to identify and map QTLs for Bakanae disease resistance using the RIL population. The whole genome was scanned for detecting QTLs using QTL IciMapping software with a LOD threshold of 2.5. The analysis of the RIL population identified four QTLs for the Bakanae disease resistance located on chromosomes 1 and 3, respectively (Fig. 3). Interval mapping (IM) identified four QTLs for Bakanae disease reaction, three mapped on chromosome 1 and one on chromosome 3 (Table 4). Among the identified QTLs, the one QTL located in the marker interval flanked by RM10153 and RM5336 on chromosome 1 with a LOD score of 15.69 explained as high as 40.59 % of phenotypic variance (PVE) for per cent seedling mortality having an additive effect of −17.63 %. Of the remaining two QTLs on chromosome 1, the QTL present in the marker interval RM9-RM11282 reported an LOD of 6.48 explaining 18.76 % of phenotypic variation with an additive effect of −11.96 %, followed by QTL located between RM10271 a LOD of 3.76 with a PVE value of 10.45 %. The QTL identified on chromosome 3 was located between the markers RM411 and RM3698 with LOD of 3.31 and explained 9.10 % of the phenotypic variation. Following the QTL naming conventions, the QTLs detected on chromosome 1 were named as *qBK1.1*, *qBK1.2* and *qBK1.3* and the QTL on chromosome 3 as *qBK3.1*.

**Table 4:** Identification of QTLs for Bakanae disease resistance using recombinant inbred lines derived from Pusa 1121/Pusa1342 cross using interval mapping (IM) and inclusive composite interval mapping (ICIM)

Method	QTL	Chr.	Position (cM)	Left Marker	Right Marker	LOD	PVE (%)	Additive effect (%)*
IM	*qBK1.1*	1	124.65	RM9	RM11282	6.48	18.76	-11.96
	*qBK1.2*	1	6.65	RM10153	RM5336	15.69	40.59	-17.63
	*qBK1.3*	1	23.65	RM10271	RM35	3.76	10.45	-9.01
	*qBK3.1*	3	41.89	RM411	RM3698	3.31	9.10	-8.34
ICIM	*qBK1.1*	1	123.00	RM9	RM11282	3.86	6.49	-7.03
	*qBK1.2*	1	5.65	RM10153	RM5336	12.07	24.74	-13.77
	*qBK1.3*	1	24.65	RM10271	RM35	2.73	4.76	-6.08

*IM* Interval mapping, *ICIM* Inclusive composite interval mapping, *QTL* Quantitative trait loci, *Chr.* chromosome, *PVE* percentage of variance explained, *additive effect has the same unit as the phenotype (% seedling mortality)

Refining the results of IM, inclusive composite interval mapping (ICIM) identified a total of three QTLs on chromosome 1, one major QTL and two minor QTLs associated to seedling mortality due to Bakanae infection. All the three QTLs were also identified by IM approach (Table 4). The major QTL, designated as *qBK1.2*, was mapped between the flanking markers RM10153 and RM5336, explaining a phenotypic variance of 24.74 %. This QTL had an additive effect of −13.8 % in mortality unit. Other two QTLs identified, *qBK1.1* and *qBK.1.3,* explained a cumulative phenotype variation of 11.25 % for seedling mortality rate among the RILs.

### *In Silico* Search for Annotated Putative Candidate Genes

The results of annotated gene search in Michigan State University –Rice Annotation Project (MSU-RAP) database using the sequences flanked by the markers RM10153 and RM5336 denoting the QTL *qBK1.2* is given in Table 5. A total of 55 genes were found annotated between the markers RM10153 and RM5336 flanking the major QTL *qBK1.2*, among which 12 genes were known to have defence related functions including *Verticillium* wilt resistance, Leucine-rich repeat family protein, putative *cf2* gene, *BRCA2* repeat family protein and unclassified resistance genes (Table 5).

**Table 5:** List of candidate genes (defense related genes) identified in the QTL mapped region *qBK1.2* on chromosome 1

Gene Locus ID	Start Position (bp)	End Position (bp)	Description or Putative Function (RAP-DB annotation)
LOC_Os01g06720	3176544	3179602	Disease resistance protein SlVe2 precursor, putative, expressed
LOC_Os01g06730	3181950	3185346	Verticillium wilt disease resistance protein, putative, expressed
LOC_Os01g06750	3196986	3200010	Verticillium wilt disease resistance protein precursor, putative, expressed
LOC_Os01g06760	3201223	3204252	Verticillium wilt disease resistance protein Ve2, putative, expressed
LOC_Os01g06790	3222303	3226257	Disease resistance protein, putative, expressed
LOC_Os01g06836	3241127	3243315	Disease resistance protein SlVe2 precursor, putative, expressed
LOC_Os01g06870	3251204	3252724	Resistance protein SlVe1 precursor, putative, expressed
LOC_Os01g06876	3256822	3257901	cf-2, putative, expressed
LOC_Os01g06890	3259438	3262454	Leucine-rich repeat family protein, putative, expressed
LOC_Os01g06900	3272839	3276416	Verticillium wilt disease resistance protein Ve2, putative, expressed
LOC_Os01g06920	3282976	3286357	Resistance protein SlVe1 precursor, putative, expressed
LOC_Os01g07110	3345203	3355066	BRCA2 repeat family protein, expressed

## Discussion

Identification of genes conferring resistance to plant diseases and the development of resistant cultivars are considered the most economic, effective and environmentally friendly measure for controlling plant diseases (Singh et al. 2011, 2012, 2013; Simko et al. 2013; Fukuoka et al. 2014; Khanna et al. 2015; Ellur et al. 2016). Researchers have made limited progress in identifying resistant sources against the Bakanae disease due to lack of reliable, reproducible and rapid assay to screen large number of germplasm against the disease. The field inoculation techniques are inherently poor in terms of reproducibility as a result of uneven inoculum distribution, interaction with other pathogens, and variations in weather and other environmental factors which may affect disease severity. This lack of information has slowed the progress of breeding programs.

In our previous study, we have developed a high throughput screening technique to screen large number of germplasm and to identify resistant sources against Bakanae disease. Using this method, we have identified Pusa Basmati 1121 as highly susceptible and Pusa 1342 as highly resistant genotypes (Fiyaz et al. 2014). A mapping population developed earlier in our lab using the above lines as parents (Amarawathi et al. 2008) was available for mapping QTLs conferring resistance to Bakanae disease. The marker polymorphism between the two parental lines was low, which can be attributed to the narrow genetic variation as the parents were *indica* ecotypes and adapted to the same rice ecosystem. Several reports are in support of low level of polymorphism between the parents in the intra-sub specific (Ali et al. 2000; Subashri et al. 2009; Gomez et al. 2010; Yadav et al. 2015) and in inter-sub specific crosses of rice (McCouch et al. 1988; Price and Tomos 1997). The polymorphic markers were filtered for their usefulness in mapping through segregation analysis because any segregation distortion can seriously affect the QTL mapping results (Xu et al. 1997).

For identifying QTLs, phenotypic measurement is very important because quantitative traits are largely affected by environment. The phenotypic frequency distributions observed in this study indicated quantitative inheritance of seedling mortality due to Bakanae disease (Fig. 2) indicating that the resistance/ susceptibility is under polygenic control. However, there was predominance of resistant progenies over susceptible ones, indicating the presence of major genetic loci in the population governing resistance. The QTL mapping technique aids to identify genetic regions affecting quantitative traits (Collard et al. 2005) that may contain or may reside proximally to the genes responsible for the trait. In this study, we have used two robust QTL mapping methods, such as IM and ICIM, of which former is a commonly used method for mapping QTLs from biparental crosses (Li et al. 2007). ICIM is an improvement over the commonly implemented composite interval mapping (CIM) algorithm, in which selection of marker is done through a stepwise regression taking into consideration of all markers simultaneously, leaving the flanking markers at the current interval. The phenotype values are adjusted for all the remaining markers retained in the regression model, and IM is then performed using the adjusted phenotype values. ICIM is therefore simple, faster and achieve model convergence efficiently, while keeping all the advantages of IM and simultaneously avoiding the complicated selection of background control markers used in CIM. ICIM is reported to have increased QTL detection power, while controlling false discovery with less biased QTL estimates than IM (Li et al. 2007).

**Fig. 4:**
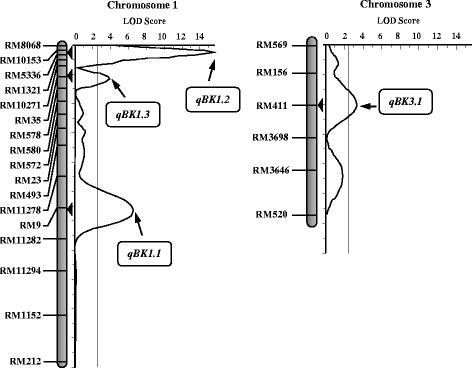
QTLs for resistance to Bakanae disease identified by interval mapping (IM) in the recombinant inbred line population derived from PB1121/Pusa1342. Arrows indicate QTL peak with their respective designations

**Fig. 5:**
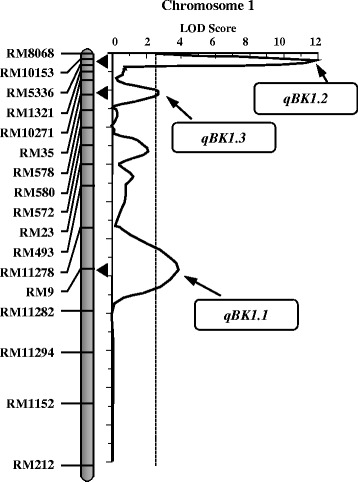
QTLs for resistance to Bakanae disease identified by inclusive composite interval mapping (ICIM) in the recombinant inbred line population derived from PB1121/Pusa1342. QTL peaks and their designations are indicated by arrows

In the current study, a total of four QTLs were identified for seedling mortality among the RILs, wherein three QTLs were detected in common by both the methods. While, IM reported four QTLs, ICIM identified only three indicating better control of QTL detection ICIM (Figs. 4 and 5). All the detected QTLs had an additive effect in the negative direction for percent seedling mortality indicating that the susceptibility alleles are contributed by the female parent, PB1121 which was highly susceptible to the pathogen and the resistant alleles by the male parent, Pusa 1342, the resistant parent. Among the QTLs identified, two QTLs *qBK1.2* and *qBK1.3* detected on the short arm of chromosome 1 are novel as there are no previous reports of detection of QTLs governing Bakanae resistance in this region of chromosome 1. The major QTL, *qBK1.2*, is a strong candidate that can be used for marker assisted introgression of Bakanae resistance. One of the minor QTLs identified in the present study, *qBK1.1* was mapped in the same genomic region in the long arm of chromosome 1, where QTL *qBK1* was reported earlier by Hur et al. (2015). The *qBK1* was mapped in the 520 kb region between RM8144 and RM11295 at corresponding physical position of 23.20 and 23.72 Mb, respectively with RM9 as the peak marker within this interval explaining 65 % of phenotypic variation. In this study, *qBK1.1* was identified between markers RM9 and RM11282 spanning from 23.32 Mb to 23.34 Mb (20 kb). Because of the co-localization of this QTL with *qBK1* and RM9 being the common marker, it is likely that *qBK1.1* identified in this study and *qBK1* identified by Hur et al. (2015), are the same QTL. Furthermore, all the QTLs identified in this study are distinct from another QTL, *qB1* identified by Yang et al. (2006), which was mapped between markers RM7180 and RM486 at corresponding physical position of 34.10 and 34.95 Mb, respectively. To our knowledge, this is the first report on two novel QTLs for resistance to Bakanae disease in rice, *qBK1.2* and *qBK1.3*, identified based on ICIM strategy, a more robust method for QTL detection.

Typical symptoms of Bakanae disease include infected plants several inches taller than the normal plants in the seedbed and field. The elongation is attributed to gibberellins (GA) and stunting to fusaric acid, both of which are produced by the fungus (Yabuta and Hayasi 1935; Takahashi et al. 1955). These symptoms are similar to seedlings treated with gibberellic acid (GA_3_) with a positive correlation existing between the GA_3_ response and the pathogenic behaviour of *F. fujikuroi* (Thakur 1974; Sunder and Satyavir 1998; Ma et al. 2008) indicating the role of gibberellins in the elongation behaviour. However, Kim et al. (2014) also reported that the GA_3_ response alone is insufficient as a direct indicator for Bakanae disease resistance, as some rice varieties display higher sensitivity to GA_3_ treatment but show resistance to Bakanae disease.

*In silico* search for putative candidate genes in a region spanning 0.26 Mb of the Bakanae resistance QTL *qBK1.2* using MSU-RGAP database identified a total of 12 genes associated with disease resistance. These genes could be possible candidates governing resistance to Bakanae disease. However, additional molecular markers needs to be screened for identifying potential recombination events which can help in higher resolution mapping to determine the gene underlying *qBK1.2* precisely. A detailed insight into such candidate genes will help in understanding the nature of interaction at molecular level governing the resistance to Bakanae disease, a step forward towards functional genomics.

## Conclusions

A novel major QTL *qBK1.2* conferring resistance to Bakanae disease has been identified in the present study using ICIM approach in a RIL population. The QTL *qBK1.2* has been putatively mapped between SSR markers RM10153 and RM5336, which can help in marker assisted introgression of this QTL for developing rice varieties with inbuilt resistance to Bakanae disease. Further, we identified two minor QTLs, which can be used to augment the resistance while developing new cultivars with improved resistance to this important disease. Fine mapping and cloning the genes for Bakanae disease resistance can not only help in developing gene based markers for use in future rice breeding but also in understanding resistance mechanisms and aid in precise selection through marker-assisted selection for development of Bakanae disease resistant cultivars.

## Methods

### Plant Material

A mapping population of 168 RILs (F_14_ generation) derived out of a cross between PB 1121, a Bakanae susceptible variety and Pusa 1342, a highly resistant variety using single seed descend method (Amarawathi et al. 2008) was used in the study. Pusa Basmati 1121, is a Basmati quality aromatic extra long slender grain variety with high alkali spreading value (low gelatinization temperature), intermediate amylose content and exceptionally high cooked kernel length, developed at the ICAR-IARI, New Delhi (Singh et al. 2002). Pusa 1342 developed from a complex pedigree involving IR8, TKM 6, Basmati 370, Pusa 1154-2 and Pusa 1201-92-1, etc., is a non aromatic elite breeding line with new plant type features, medium grain length, high amylose content, low alkali spreading value and medium kernel elongation upon cooking. The parent genotypes were identified from a germplasm screening study in which 92 genotypes were studied for Bakanae response on artificial inoculation (Fiyaz et al. 2014). Well before this screening experiment, the RIL mapping population, between PB1121 and Pusa 1342 was already available at the Division of Genetics, ICAR-IARI, New Delhi. This RIL population was developed by Amarawathi et al. (2008) for mapping quality related traits in Basmati.

### Phenotyping of F_14_ RIL Population

The experiment was conducted in the glasshouse of ICAR-IARI, New Delhi, India. The reactions of 168 RILs to Bakanae disease along with their parents were assessed by artificial inoculation of a virulent isolate of *F. fujikuroi*, F250 (NCBI Gene bank accession number - KM50526) through a high throughput screening protocol developed by Fiyaz et al. (2014). In this method, the disinfected seeds of each of the 168 RILs were soaked in 10 ml of inoculum suspension containing 1×10^6^ spores ml^-1^ for 24 h at room temperature. Uninoculated seeds were disinfected before soaking in sterile water for 24 h. Inoculated and uninoculated seeds were sown in 7×14 plastic pottrays (one pottray per RIL/ 98 seeds per pottray) containing autoclaved mixture of soil and sand in the ratio of 3:1. The glass house temperature was maintained at 30/25(±3) °C day/night temperature regime, 60/80(±10) % day/night relative humidity, with natural sunlight. Pottrays with seedlings were watered every day to keep them in saturated condition. No additional fertilisation was done in pottrays. Post inoculation, the seedlings were carefully observed for the symptoms of Bakanae infection. Data on number of seedling mortality were observed everyday upto 15 days (Wulff et al. 2010). The phenotyping of the RILs was repeated six times to avoid any error in phenotyping. Due to uneven elongation of RIL seedlings under Bakanae infection, the seedlings elongation trait was not used for mapping studies. Since there was no seedling mortality under uninoculated conditions, among both the parents and the RILs, the statistical analysis of the seedling mortality was confined only to inoculated system, and the data were analysed using standard procedures.

### Construction of Molecular Linkage Map

A total of 732 simple sequence repeat (SSR) markers were used for the parental polymorphism survey between the resistant (Pusa 1342) and susceptible (PB 1121) parent. The total genomic DNA of the RILs was extracted with slight modification to the protocol of Murray and Thompson (1980). Polymerase chain reaction (PCR) was performed in a thermal cycler (Applied Biosystems® Veriti®, California, USA) using a total reaction volume of 10 μl. This contained 30 ng of template DNA, 5 pmol of each primer (synthesized from Sigma Inc., St. Louis, MO, USA), 1.5 mM MgCl_2_, 0.2 mM dNTPs (MBI, Fermentas, Vilnius, Lithuania) and 0.5 U of Taq polymerase (Bangalore Genei, Bangalore, Karnataka, India). Polymerase chain reaction comprised one cycle of denaturation at 95 °C for 5 min, followed by 35 cycles at 95 °C for 30 s, 55 °C for 30 s and 72 °C for 1 min, with a final extension of 72 °C for 7 min. The amplified products were resolved on 3.5 to 4.0 % Metaphor^TM^ Agarose gel containing 0.1 mg/ml of ethidium bromide (Amresco, Solon, OH, USA) along with a DNA size standard 50 bp ladder (MBI, Fermentas) and visualized on ultraviolet transilluminator (Gel Doc^TM^ XR + Imager, Bio-Rad Laboratories Inc., U.S.A). The polymorphic SSR markers identified from the parental polymorphism survey were used for genotyping of 168 RIL population along with parental lines. Data generated after genotyping of 168 RILs by polymorphic SSR markers were tested using the *χ*^2^ goodness of fit test for checking segregation distortion. A linkage map was constructed by using the linkage mapping function implemented in the QTL IciMapping software (Meng et al. 2015). The map distances were calculated based on Kosambi’s mapping function (Kosambi, 1944).

### QTL Mapping

QTL mapping was performed by interval mapping (IM) and inclusive composite interval mapping (ICIM) functions implemented in the QTL IciMapping v3.3 (www.isbreeding.net). Two or more closely linked markers that showed significant association were assumed to identify the same QTL. To determine the precise location of the putative QTLs, interval mapping and inclusive composite interval mapping functions were used. Inclusive composite interval mapping (Wang 2009) was used to estimate QTL effects such as log-likelihood ratio (LOD) score, phenotypic variation explained (PVE), and additive effect of the QTL loci. The threshold LOD value was determined by a permutation test involving 3000 runs at a significance level of *p* = 0.05. The LOD test statistic used was -2ln (L_0_/L_1_), where L_0_/L_1_ is the ratio of the likelihood under the null hypothesis (there is no QTL in the interval) and the alternative hypothesis (there is a QTL in the interval). The QTLs were deemed to exist only at positions where an LOD score exceeded the corresponding significant threshold. Estimation of position, genetic effects and phenotypic variation percentage of the QTLs were done at the significant LOD peak in the region under consideration. QTL nomenclature followed that described by McCouch et al. (1997).

### *In Silico* Search for Annotated Putative Candidate Genes

For the identified major QTL, that explained high level of phenotypic variation, an *in-silico* search was done for the chromosomal interval based on the physical location of the flanking markers for the presence of candidate defense responsive genes associated with Bakanae disease resistance using Michigan State University –Rice Annotation Project (MSU-RAP) database version 7.0 (Kawahara et al. 2013) available at http://rice.plantbiology.msu.edu. The hypothetical and expressed genes present in chromosome 1 starting from 3099428 to 3367663 bp region was collected as the list of annotated genes in the QTL region. The annotated putative genes were further explored for their known role in defense mechanisms including disease resistance.
